# Towards Understanding the Task Dependency of Embodied Language Processing: The Influence of Colour During Language-Vision Interactions

**DOI:** 10.5334/joc.135

**Published:** 2020-10-22

**Authors:** Falk Huettig, Ernesto Guerra, Andrea Helo

**Affiliations:** 1 Max Planck Institute for Psycholinguistics, NL; 2 Centre for Language Studies, Radboud University, NL; 3 Centro de Investigación Avanzada en Educación, Instituto de Educación, Universidad de Chile, CL; 4 Departamento de Fonoaudiología, Universidad de Chile, CL; 5 Departamento de Neurociencias, Universidad de Chile, CL

**Keywords:** Embodied cognition, Eye movements, Visual world paradigm

## Abstract

A main challenge for theories of embodied cognition is to understand the task dependency of embodied language processing. One possibility is that perceptual representations (e.g., typical colour of objects mentioned in spoken sentences) are not activated routinely but the influence of perceptual representation emerges only when context strongly supports their involvement in language. To explore this question, we tested the effects of colour representations during language processing in three visual-world eye-tracking experiments. On critical trials, participants listened to sentence-embedded words associated with a prototypical colour (e.g., ‘…spinach…’) while they inspected a visual display with four printed words (Experiment 1), coloured or greyscale line drawings (Experiment 2) and a ‘blank screen’ after a preview of coloured or greyscale line drawings (Experiment 3). Visual context always presented a word/object (e.g., frog) associated with the same prototypical colour (e.g. green) as the spoken target word and three distractors. When hearing *spinach* participants did not prefer the written word *frog* compared to other distractor words (Experiment 1). In Experiment 2, colour competitors attracted more overt attention compared to average distractors, but only for the coloured condition and not for greyscale trials. Finally, when the display was removed at the onset of the sentence, and in contrast to the previous blank-screen experiments with semantic competitors, there was no evidence of colour competition in the eye-tracking record (Experiment 3). These results fit best with the notion that the main role of perceptual representations in language processing is to contextualize language in the immediate environment.

## Introduction

The notion that simulations of sensory, motor and affective representations underlie understanding of spoken and written language comprehension, typically referred to as *embodiment* or *embodied language processing* (e.g., Barsalou, 1999; Fischer & Zwaan, 2008; Meteyard, Cuadrado, Bahrami, & Vigliocco, 2012), has become an influential theoretical account of language processing. Yet, despite its increasing popularity, many theoretical and empirical questions about embodiment remain hotly debated (e.g., Mahon & Caramazza, 2008). Ostarek and Huettig (2019) recently have put forward six challenges that embodiment research has to face in order to become a fully plausible account of language processing and cognition more generally. We will briefly discuss why it is important that future research directly addresses these challenges. We will then explain how the present study addresses one of these challenges, namely, to more fully understand the task dependency of embodied language processing.

More than 20 years after Barsalou’s (1999) seminal paper on perceptual symbol systems, embodiment has moved in status from an outlandish proposal advanced by a fringe movement in psychology to a mainstream position adopted by large numbers of researchers in the psychological and cognitive (neuro)sciences (e.g., Anderson et al., 2015; Fernandino et al, 2016; Kaschak & Glenberg, 2000; Pulvermüller et al., 2005; Vukovic et al., 2017; Willems et al., 2011; Zwaan, 2014). We conjecture that the reason that despite a wealth of research (see Barsalou, et al., 2003; Fischer & Zwaan, 2008; Glenberg, 2010; Meteyard et al., 2012; for comprehensive summaries) the key issues (e.g., as outlined by Mahon & Caramazza, 2008) have not been resolved is that most studies have not directly (enough) addressed the open questions. This however is of crucial importance to make progress in our understanding of embodied cognition as a serious candidate theory of human information processing.

Ostarek and Huettig (2019) posed six challenges directed at overcoming this theoretical impasse. The first challenge for embodiment research is to develop paradigms that directly probe for the sensory, motor, and affective representations that are assumed to underlie language processing. Continuous flash suppression (CFS), for instance, appears to be a useful technique in this regard because it allows to investigate how spoken language modulates detection of visual features of concurrently presented (and visually suppressed) pictures. Evidence from this paradigm suggests that semantic processing of spoken words can (at least in principle) involve (visual) perceptual processes (Lupyan & Ward, 2013; Ostarek & Huettig, 2017a). A second challenge is to probe the causality of simulations in language processing directly. Looking at displays with visual noise, for example, has been shown to interfere with participants’ concurrent processing of spoken words in a concreteness judgment task in which visual information is relevant but not in lexical decision or word class judgment tasks (Ostarek & Huettig, 2017b). Looking at displays with visual noise did also not interfere in a spoken version of the classic shape match effect in sentence-picture verification tasks (Ostarek, Joosen, Ishag, De Nijs, & Huettig, 2019), an effect that is often regarded as as a seminal demonstration of embodied language processing (e.g., Zwaan, Stanfield, & Yaxley, 2002). A third challenge is to be explicit about the direction and timing of hypothesized experimental effects before the experiment is conducted (we will discuss this further in the general discussion of this paper with a focus on inconsistent colour effects in the sentence-picture verification paradigm). Another challenge is to work towards developing an all-encompassing theory of of embodied language processing that provides a convincing account not only for the processing of spoken words and sentences that refer to concrete objects but also abstract ideas and events (but see Guerra & Knoeferle, 2014, 2017). A fifth challenge is to assess embodiment using a diverse set of methods (including novel methods) that converge on similar conclusions.

A sixth challenge, and the one we address in the present study, is to more fully understand the task dependency of embodied language processing. It is important to note here that some previous studies have focused on the notion that different situations may make different aspects of language (e.g., different aspects of meaning) contextually relevant (e.g., Estes & Barsalou, 2018). Other studies have focused on strong interpretations of embodiment and have proposed that sensory representations are *routinely* activated to influence language processing (e.g., Wassenburg & Zwaan, 2010). The notion of routine activation is not very well supported by current experimental evidence. Rommers, Meyer, and Huettig (2013) investigated this issue by presenting participants with sentences that implied that an object mentioned had a specific shape or orientation. Participants were then asked to either name a picture of that object (Experiments 1 and 3) or decide whether the object had been mentioned in the sentence (Experiment 2). Orientation information did not reliably influence performance in any of the tasks. Shape representations influenced performance most strongly in sentence-picture verification (i.e. when participants were asked to compare a sentence with a picture) or when they were explicitly asked to use mental imagery while reading the sentences. This study thus suggested that implied visual information often does not contribute substantially to the comprehension process during normal reading. Nevertheless, the notion that (perceptual) simulations occur in some (but not all) situations requires further empirical work. Thus, in this paper we ask: Are perceptual representations, such as the typical colour of objects mentioned in spoken sentences, activated routinely in language processing, or alternatively, does the influence of perceptual representation emerge only when context strongly supports their involvement in language?

We tested the effects of colour representations during language processing in three visual-world eye-tracking experiments. We believe that this method (i.e. the visual world paradigm, Cooper, 1974; Tanenhaus, Spivey-Knowlton, Eberhard, & Sedivy, 1995) is well-suited to investigate this issue but it is a method that has not frequently been used to explore questions about embodiment (but see Kamide, Lindsay, Scheepers, & Kukona, 2016; Lindsay, Scheepers, & Kamide, 2013; Myung, Blumstein, & Sedivy, 2006; Myung, Blumstein, Yee, Sedivy, Thompson-Schill & Buxbaum, 2010, for notable exceptions). We therefore discuss briefly the main features of the method.

The visual-world eye-tracking method makes use of the tight connection between spoken language processing and visual processing that has been established in a great number of studies (see Huettig, Rommers, & Meyer, 2011; Knoeferle & Guerra, 2016; Magnuson, 2019, for reviews), in particular, when participants hear a word that refers to a visual object or printed word in their concurrent visual environment they quickly (and semi-automatically, cf. Mishra et al., 2013) direct their eye gaze to objects or printed words which are similar (e.g. semantically or visually) to the heard word. Data analyses in visual-world studies focus on the question of how likely the participants are to look at specific regions of interest at different times during a trial. By averaging across trials and participants, it can be computed how likely listeners are, on average, at a given moment in time, to look at each of the areas of interest. Based on such data, inferences about the fine-grained time course of the underlying cognitive processes can be drawn. Needless to say, the paradigm has also some important short-comings. Speech presented must be related to relevant visual input despite the strong eye gaze - cognitive processing link. The paradigm does not allow to determine the importance of perceptual representations in the absence of visual input. However, this eye-tracking paradigm is particularly suited to provide further insight to investigate the task dependency of embodied language processing because the availability of task-relevant linguistic and visual input can be systematically manipulated.

A previous study by Huettig and Altmann (2011) is particularly relevant in this regard. In their visual-world experiments, participants heard sentences that contained words whose concepts are associated with a diagnostic colour (e.g., “*spinach*”; which is typically green) while their eye movements were measured to (i) objects associated with a diagnostic colour but shown in greyscale (e.g., a greyscale line drawing of a frog), (ii) objects associated with a diagnostic colour but shown in a possible but atypical colour (e.g., a colour photo of a yellow frog), and (iii) objects not associated with a diagnostic colour but shown in the diagnostic colour of the target concept (e.g., a green blouse; blouses are not associated with the colour green). They found that eye gaze was mostly driven by the perceived surface attributes of the visual objects and not the stored knowledge about the typical colour of the object. Their experiments also suggested that conceptual category information was the main determinant of eye gaze when both conceptual category and surface colour competitors were shown in the same visual displays.

## Current study

Huettig and Altmann’s (2011) study thus suggested a strong task dependency of embodied language processing. Crucially, the nature of the visual environment (in particular the explicit availability of colour in the surroundings) appeared to be of prime importance for the access and use of ‘language-derived’ colour representations. Here we tested the influence of colour representations during language processing further in three different visual-world situations. As with Huettig and Altmann (2011), we used a look and listen task which previously has been shown to be sensitive to such relationships between spoken words and visual items. In Experiment 1, on experimental trials, participants listened to (Dutch) sentences containing a critical target word associated with a prototypical colour (e.g., ‘…spinach…’) as they inspected a visual display with four words printed in black font. One of the four printed words was associated with the same prototypical colour (e.g. green) as the spoken target word (e.g. FROG). On experimental trials, the spoken target word did not have a printed word counterpart (SPINACH was not present in the display). In target trials (70% of trials) the target was present in the display. Experiments using the printed word version (Huettig & McQueen, 2007; Viebahn & McQueen, 2007) of the visual world paradigm have shown that printed words which are orthographically (Salverda & Tanenhaus, 2010), phonologically (Huettig & McQueen, 2007; Viebahn & McQueen, 2007), and semantically (Huettig & McQueen, 2011) similar to concurrently heard spoken words attract increased visual attention. A surprising previous finding (Huettig & McQueen, 2011) was that semantic relationships (pen/desk) but not visual shape similarity (pen/cigarette, both have a similar global shape) results in increased eye gaze to the competitors with printed word displays. The absence of the shape competitor effects with printed words (in contrast to robust phonological, orthographic, and semantic competitor visual world effects with printed words) does not fit well with notions that perceptual representations are routinely activated during language processing. The present Experiment 1 was another test with a different perceptual feature to explore this. We tested whether such an effect also occurs for printed words whose referent is related in prototypical colour (“spinach”, FROG). If colour information is routinely accessed and simulated such a visual world effect should occur.

In Experiment 2 we investigated the effect of colour with visual objects. The line drawings of the objects were presented instead of the printed words. We used a within-participants counter-balanced design and alternated colour and greyscale trials randomly throughout the experiment to direct the attentional focus of the participants towards the colour features. The presence and absence of colour in individual trials was therefore apparent to the participants. If colour information is routinely accessed and simulated, then increased looks to colour competitors should occur in both colour and greyscale trials. In contrast, if the influence of perceptual representations is depending on a (visual) context which strongly supports their involvement in language, then increased looks to colour competitors should occur in colour trials but *not* in greyscale trials.

Finally, in Experiment 3, we replaced the object visual display at the sentence onset with a blank screen. This was done to investigate whether the continued presence of colour in the immediate visual environment is a necessary condition for the observation of colour-mediated eye movements. Previous studies with such a blank screen paradigm have shown that people direct eye movements towards locations that were previously occupied by target objects even when it is completely unnecessary for the task (Spivey & Geng, 2001; Altmann, 2004). We reasoned that if colour information is routinely accessed and simulated, then we should observe evidence of it with the blank screen method. Such an outcome is expected because previous research has shown that participants form episodic memory traces in which the visual object identities are bound to their previous locations. Once spoken language refers to the previously fixated related object, then its original location is retrieved and tends to trigger an eye movement to that location on the blank screen (Ferreira, Apel, & Henderson, 2008; Richardson, Altmann, Spivey, & Hoover, 2009). Experiment 3 and the blank screen paradigm was therefore our final test exploring the task dependency of activation and simulation of colour information.

## Experiment 1

### Method

#### Participants

Thirty native speakers of Dutch with normal or corrected-to-normal vision from the MPI for Psycholinguistics participant pool took part in the experiment. All participants gave informed consent and received monetary compensation for the participation.

#### Materials and design

**Figure 1:**
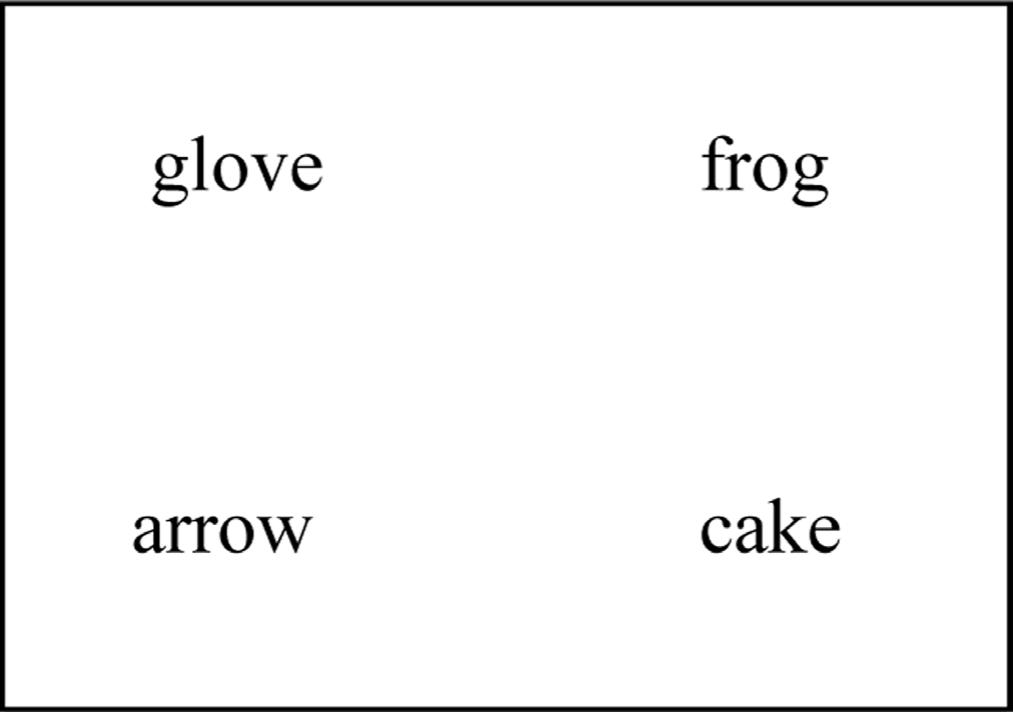
(Translated) visual context example for an experimental trial in Experiment 1.

In our first visual-world eye-tracking experiment, the visual display consisted of four written words (see, e.g., McQueen & Viebahn, 2007), each presented in the middle of one out of four fixed locations from a 5 × 5 invisible grid (Figure 1). For the linguistic materials, we used 14 experimental and 28 control sentences (translated to Dutch form from Huettig & Altmann, 2011). Both experimental and control sentences were recorded by a female native speaker of Dutch in a sound-damped booth. In all trials, a critical word was embedded in a neutral sentence that did not allow participants to make predictions about upcoming words. The average word onset was three seconds after sentence onset.

On experimental trials, the critical word referred to an object strongly associated with a given colour (e.g., ‘The man thought about it for a while and then he looked at the spinach and decided to try out the recipe’; here ‘spinach’ is associated with the colour green). Importantly, on those trials one of the printed words was a concrete noun also associated with the same colour (e.g., the printed word ‘frog’ is a colour competitor to the spoken word ‘spinach’; see Table 1 and Huettig & Altmann, 2011, for further details), hence we call the experimental condition *competitor*. In control trials instead, the critical word did not refer to objects associated with a particular colour and always appeared printed in the visual display. Thus. we will refer to trials in this condition as *target*.

**Table 1:** List of linguistic materials used in all three experiments.

Target word	Colour association	Competitor word

grasshopper	green	broccoli
frog	green	spinach
lettuce	green	emerald
celery	green	turtle
tree	green	lizard
leaf	green	crocodile
cherry	red	lipstick
tomato	red	brick
strawberry	red	fox
lobster	red	raspberry
lips	red	rose
banana	yellow	canary
lemon	yellow	dandelion
corn	yellow	chick

A repeated measures within-participants design was used across the two conditions. A single experimental list, which randomly combined both the 14 experimental and 28 control trials, was presented to each participant.

#### Procedure

Participants’ eye-movements were recorded using an Eye-link 1000 Tower Mount (SR Research) as they heard spoken sentences and inspected the corresponding visual displays. At the beginning of the session a calibration procedure was carried out, the procedure was repeated every four trials if necessary. Participants were instructed to listen to the spoken sentences attentively. They were told that they could inspect the visual context freely, but they should maintain their eyes on the monitor during the experiment. No explicit responses or other tasks were requested. Such a task situation has been extensively studied and successfully been used in a great number of studies (Huettig, Rommers, et al., 2011, for further discussion). On each trial, participants observed a visual context with four written words for one second before the sentence started.

#### Data Analysis

Using the Data Viewer software (SR Research), four regions of interest (ROI) were defined surrounding each of the four written words on the display. Participants’ fixations durations and locations on the display were summarized using the same software. Thus, fixations to the critical printed word (colour competitors and target) could be individualized, as well as those fixations to the distractor printed words. We analysed the first 1000 ms after the onset of the critical word for each sentence on each condition. For each time step of one ms in this time window, a value of 1 was given to the ROI that the participant was looking at, while all other ROIs received a 0. If no ROI was fixated in a given time window, a 0 was assigned to all four ROIs. This procedure was conducted per participant per trial. We then aggregated the data by averaging every 50 milliseconds again per participant, trial, and ROI. The mean fixation proportion and the 95% confidence intervals (CI) adjusted for within-subject designs (see Morey, 2008) were calculated for each 50 ms time window within the critical time window for the critical written word and the average distractor (cf. Huettig & Janse, 2016; Huettig & Guerra, 2019). The proportion of fixations to the average distractor was computed as the mean fixation proportion of the three non-critical printed words in the display.

Inferential analysis was implemented through growth curve analysis on empirical logit transformations of the proportion of fixations (Barr, 2008, 2013; Mirman, Dixon, & Magnuson, 2008; Mirman, 2014). Growth curve analysis allows to directly predict time in a single analysis using orthogonal higher-order polynomials as predictors of the non-linear changes of proportion of looks over time. Before analysis, we transformed fixation proportions to empirical logits for each time window, scaling binary data to a continuous variable (Barr, 2008; Mirman, 2014). Afterwards, four models that differed only in their number of polynomial terms (from linear to quartic terms in ascending cumulative order) were compared using the *anova* function in R. All four models had the polynomial terms as fixed effects, as well as the interaction between object (target vs. average distractor) and trial type (competitor vs. target) and their interaction with the polynomials. The random structure of the models included cross-random intercepts for participants and items, and random slopes for each polynomial predictor. The models did not include random correlations between random effects to facilitate convergence (see Barr et al., 2013). Following Baayen (2008), we use a *t*-value > |2| criteria to estimate significance. Data files and script are available at https://osf.io/853nk/.

#### Results

**Figure 2:**
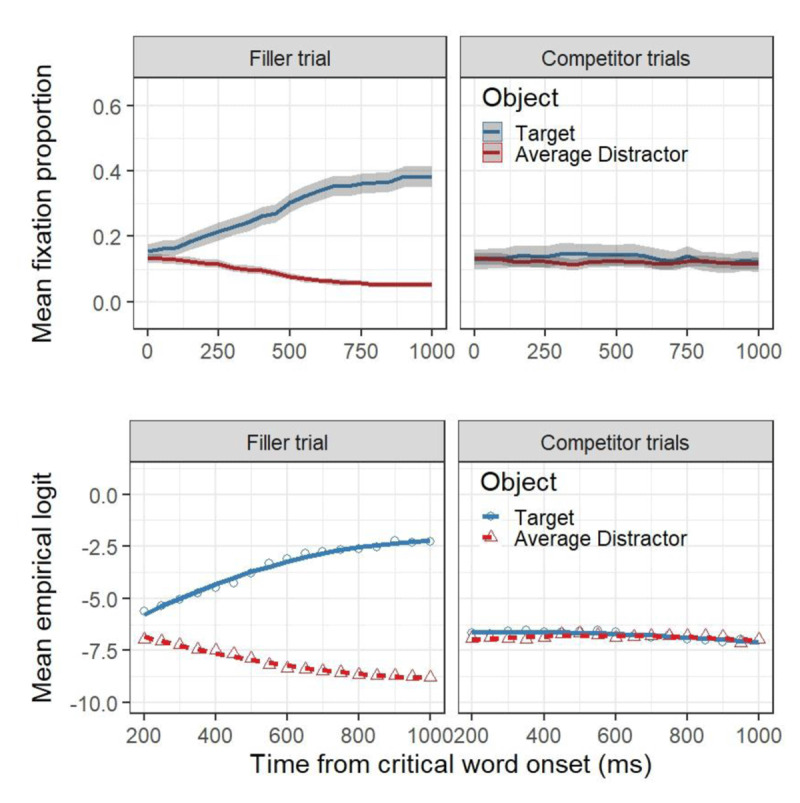
Upper panels: Mean fixation proportion as a function of trial type, aggregated in time steps of 50 ms. The plots are time-locked to the onset of the critical word. Blue and red lines represent looks to the target and the average distract, respectively and the shaded areas represent within-subject adjusted 95% confidence levels, calculated by participant. Lower panels: GCA model fit (lines) of empirical logit (points) to the target and the average distractors as a function of trial type and experimental condition.

Figure 2 presents the the proportion of eye fixations (upper panels) and the GCA model fit of empirical logits (lower panels) to critical and average distractor words over time for both the target (on the left) and the competitor conditions (on the right). Blue lines represent looks to target words, while red lines represent looks to the average distractors. Finally, in the upper panels the shaded grey areas surrounding the lines represent the within-subject adjusted CIs for the target words and average distractor word. GCA model comparison resulted in the selection of a model that included only two orthogonal polynomial terms (i.e., linear and quadratic, χ^2^ = 77.25, *df* = 12, *p* < .001).

As shown in Figure 2 (upper-left panel), target trials elicited a rapid and extended effect on the proportion of fixation towards the critical printed word relative to the average distractor words. By contrast, competitor trials elicited no changes on the proportion of fixation towards critical printed words relative to the average distractor words. The results of the GCA model are presented in Table 2, showing reliable main effects of object, trial type as well as their interaction. This reflects both the large preference for the referent in the target trials, as well as the absence of a preference for the competitor on competitor trials. Moreover, we observed interaction effects between the linear term and object, between the linear term and trial type, and the quadratic term and object. Finally, we observed three-way interactions between each of the polynomial terms, object and trial type. Based on the visual representation of the models (Figure 2, lower panels), this can be interpreted as the target object on target trials having a more quadratic time course relative to the other three objects, which appear to have a more linear time course.

**Table 2:** Main and interaction effect in the quasi-logistic GCA mixed model analysis in Experiment 1.

	Estimate	se	t

(Intercept)	–6,36	0,29	–21,61*
Linear	0,34	0,42	0,81
Quadratic	–0,22	0,23	–0,96
Object	–1,16	0,03	–39,21*
Type	0,55	0,03	16,62*
Object * Type	–1,11	0,03	–37,59*
Linear * Object	–1,49	0,34	–4,40*
Linear * Type	0,66	0,29	2,30*
Quadratic * Object	0,40	0,19	2,07*
Quadratic * Type	–0,03	0,16	–0,21
Linear * Object * Type	–1,99	0,13	–14,82*
Quadratic * Object * Type	0,41	0,13	3,24*

#### Discussion

In Experiment 1 participants listened to sentences containing a critical target word associated with a prototypical colour (e.g. ‘…spinach…’) as they inspected a visual display with four words printed in black font. The critical manipulation was that the spoken target word did not have a printed word counterpart (SPINACH was not present in the display) but that one of the four printed words was associated with the same prototypical colour (e.g. green) as the spoken target word (e.g. FROG). This target absent–design has been extensively studied and successfully employed in many studies (Huettig & McQueen, 2007; Viebahn & McQueen, 2007; see Huettig, Rommers, et al., 2011, for further discussion). The colour competitors were not looked at more than the distractors. In the target trials, however, which were 70% of the trials, the target was present in the display and attracted robustly more overt visual attention than the unrelated distractors.

These findings are in line with a previous study by Huettig and McQueen (2011). They observed in four experiments that semantic relationships (pen/desk) but not visual shape (pen/cigarette, both have a similar global shape) resulted in increased overt attention to the competitors with printed word displays. Huettig and McQueen (2011) interpreted their results as suggesting that information about the typical shape of visual objects is not retrieved rapidly or used to guide eye gaze around the display. Importantly, they argued that it is not the case that retrieval of shape information is blocked because eye gaze is driven by other types of information (e.g., by phonological, orthographic, or semantic matches, Huettig & McQueen, 2007) that match between spoken language and printed word displays. It seemed the display itself (i.e., printed words rather than pictures) signalled that information about the typical shape of visual objects should not be retrieved rapidly or used to guide eye gaze.

We believe that the findings of the present Experiment 1 (no effect of colour relations with printed word displays) can be explained in similar ways. The lack of a preference for the colour competitors was observed because printed words induce an implicit bias against the rapid online use of (conceptual) colour information. Note that this bias is implicit in the sense that participants are not explicitly choosing to ignore stored colour knowledge. Rather, the bias is driven implicitly by the nature of the input. While information about the colour of objects is present in (coloured) picture displays and is used immediately to direct eye gaze around such displays, it must be accessed from long-term memory when printed word displays are used. Our experiment (in line with Huettig & McQueen, 2011) suggests there is no fast and efficient retrieval of colour (and object shape) information when someone sees an array of printed words.

Experiment 1 thus suggests that perceptual representations such as the typical colour of objects mentioned in spoken sentences are not activated routinely. It demonstrates that different types of visual information (e.g., pictures or printed words) induce implicit biases toward particular modes of processing during language-mediated visual search.

## Experiment 2

In Experiment 2 we replaced the printed words with line drawings of the objects. In order to direct the attentional focus of our participants toward colour features we used a within-participants counter-balanced design and alternated colour and greyscale trials randomly throughout the experiment. Therefore, on one trial our participants heard a word such as ‘spinach’ and saw a frog (coloured in green) in the visual display. On the next trial however they saw a banana (in greyscale) on hearing ‘canary’ (bananas and canaries are typically yellow), and so forth. The presence (or absence) of colour was thus a salient property of the experiment. If colour information is routinely accessed and simulated we would expect increased looks to colour competitors to occur in both colour and greyscale trials. If, on the other hand, the influence of perceptual representations emerges only when context strongly supports their involvement in language we expect increased looks colour competitors in colour trials but *not* in greyscale trials.

### Method

#### Participants

Twenty-eight new members of the participant panel of the MPI for Psycholinguistics were paid for participation. All were native speakers of Dutch and had either uncorrected vision or wore soft contact lenses or glasses. All participants gave informed consent.

#### Materials and design, procedure and data analysis

**Figure 3:**
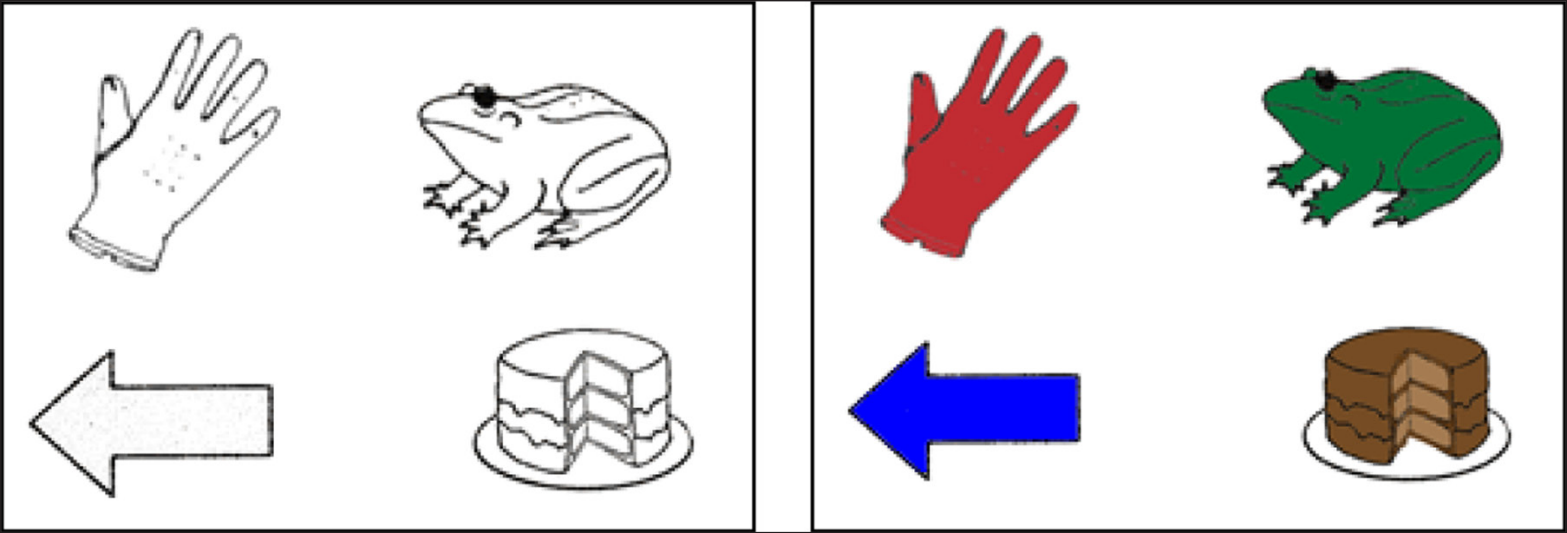
Visual display example for experimental trials as a function of experimental condition in Experiment 2.

Acoustic materials were the same as those used in Experiment 1. In the visual display, however, the four written words were replaced by visual depictions of the objects referred to by the words. Moreover, the pictures could appear in either of two experimental conditions; a colour condition or a greyscale condition. In the greyscale condition the visual displays consisted of line drawings presented in black-and-white, while in the colour condition the line drawings were coloured. In the colour competitor condition, the critical picture (e.g., *frog*) was always coloured in the prototypical colour associated with the critical sentence-embedded spoken word (e.g., green, see Table 1). The three distractors were coloured uniquely but in an appropriate manner. As in Experiment 1, these pictures were centred to one of the four fixed locations from a 5 × 5 invisible grid (Figure 3). The same 28 target trials as in Experiment 1 were presented, and the accompanying visual context was also changed to line drawings. Half of them were presented in greyscale and in the other half, they were coloured.

A within-participants counter-balanced design crossed the two experimental manipulations through two experimental lists with seven competitor trials in the colour condition and seven competitor trials in the greyscale condition. The spoken sentences were identical across the two lists and the visual displays (colour or greyscale) were rotated across them. Thus, participants’ saw either the colour version or the greyscale version of a particular trial. The 28 target trials were also split between colour and greyscale visual display in equal numbers, yet these trials were maintained constant across lists. The procedure for the experiment and the data analysis approach in Experiment 2 were identical to Experiment 1, except for the number of factors in the GCA analysis. Experiment 2 included (in addition to the object and trial type) the colour manipulation (coloured vs. greyscale) as a fixed effect as well as the corresponding interactions with the other predictors. All other aspects of the analysis were kept the same. Data files and script are available at https://osf.io/853nk/.

#### Results

**Figure 4:**
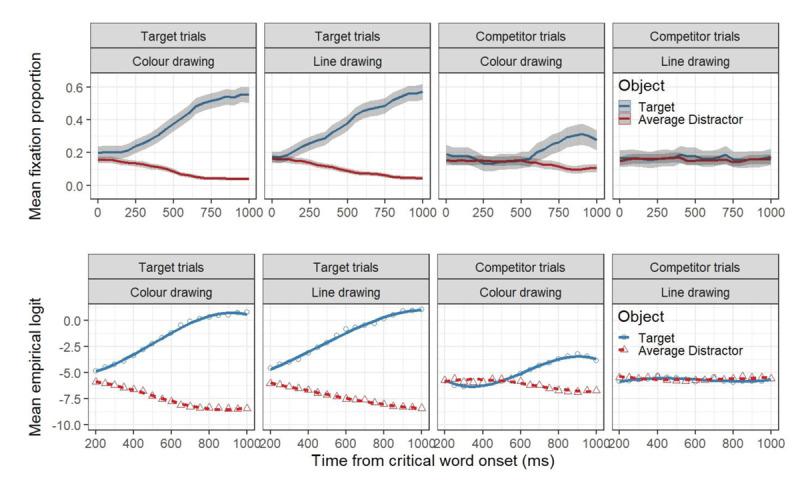
Upper panels: Mean fixation proportion as a function of trial type, aggregated in time steps of 50 ms. The plots are time-locked to the onset of the critical word. Blue and red lines represent looks to the target and the average distractors, respectively and the shaded areas represent within-subject adjusted 95% confidence levels, calculated by participants. Lower panels: GCA model fit (lines) of empirical logits (points) to the target and the average distractors as a function of trial type and experimental condition.

Figure 4 presents the time course graphs of 1000 ms for the proportion of fixations towards critical and average distractor objects (upper panels), as well as the GCA model fit of empirical logits (lower panels), as a function of trial type (target vs. competitor) and colour (colour vs greyscale) experimental conditions. Blue lines depict changes in participants’ looks towards the target objects, while red lines do so for the average distractors in all graphs. Shaded grey areas around fixation proportion lines represent CIs. The results from inferential analysis are presented in Table 3. The GCA model comparison resulted in the selection of a model that included three orthogonal polynomial terms (i.e., linear, quadratic and cubic, χ^2^ = 31.54, *df* = 18, *p* < .05).

**Table 3:** Main and interaction effect in the quasi-logistic GCA mixed model analysis in Experiment 2.

	Estimate	se	t

(Intercept)	–5,00	0,22	–22,57*
Linear	1,40	0,47	3,01*
Quadratic	–0,09	0,18	–0,51
Cubic	–0,20	0,13	–1,49
Object	–1,62	0,03	–53,21*
Type	0,53	0,03	15,42*
Colour	–0,01	0,03	–0,23
Object * Type	–1,34	0,03	–44,20*
Object * Colour	–0,17	0,03	–5,59*
Type * Colour	–0,05	0,03	–1,78
Linear * Object	–3,53	0,31	–11,56*
Linear * Type	0,78	0,28	2,83*
Linear * Colour	0,32	0,28	1,11
Quadratic * Object	0,37	0,14	2,64*
Quadratic * Type	–0,16	0,14	–1,10
Quadratic * Colour	0,00	0,17	–0,01
Cubic * Object	0,42	0,13	3,39*
Cubic * Type	0,00	0,13	0,02
Cubic * Colour	–0,17	0,13	–1,26
Object * Type * Colour	0,16	0,03	5,28*
Linear * Object * Type	–2,10	0,13	–16,72*
Linear * Object * Colour	–0,87	0,13	–6,92*
Linear * Type * Colour	–0,40	0,14	–2,90*
Quadratic * Object * Type	0,47	0,13	3,74*
Quadratic * Object * Colour	–0,06	0,13	–0,46
Quadratic * Type * Colour	0,02	0,13	0,13
Cubic * Object * Type	0,08	0,13	0,63
Cubic * Object * Colour	0,41	0,13	3,27*
Cubic * Type * Colour	0,17	0,13	1,30
Linear * Object * Type * Colour	0,66	0,13	5,27*
Quadratic * Object * Type * Colour	0,32	0,13	2,51*
Cubic * Object * Type * Colour	–0,19	0,13	–1,52

As can be seen in the upper panel of Figure 4, target trials in both the colour trials and the greyscale trials, revealed a rapid preference for the target object relative to the average distractor. In competitor trials, however, the target object (e.g., *frog* when hearing ‘spinach’) received more attention relative to the average distractor in the colour trials, but not for the greyscale trials.

Table 3 shows main effects of the linear term, object and trial type, showing that fixation proportions in general tend to linearity over time, that the targets are overall preferred over the average distractors, and that the magnitude of that preference is significantly larger in target trials compared to competitor trials. Moreover, we found an interaction between object and trial type, and between object and colour condition, reflecting the larger preference for the target object on target trials as well as the absence of this effect in line-drawing trials on competitor trials. Similarly, we found interaction effects between each polynomial and object, as well as between the linear term and trial type. These effects can be interpreted as reflecting an overall tendency to linearity for the average distractor and to greyscale trials, while the target objects and the colour trials exhibit a more cubic shape (see Figure 4, lower panels).

More critically, we found a reliable three-way interaction between object, trial type and the colour condition. This interaction clearly reflects the distinctive effect of coloured images on the preference for the target on competitor trials and target trials: while in target trials the target is clearly preferred over the average distractor independently of the coloured condition, in competitor trials the target is preferred only when the visual display is coloured. We also observed three-way interactions between the linear term, object and trial type, the linear term, object and colour condition, and between the linear term, trial type and colour condition. Similarly, we observed reliable three-way interactions between the quadratic term, object and type, as well as the cubic term, object and colour. Finally, two reliable four-way interaction were observed. The linear term and the quadratic term both interacted with object, trial type and colour condition.

#### Discussion

In order to direct the attentional focus towards colour information Experiment 2 used a within-participants counter-balanced design with randomly varied (alternated) colour and greyscale trials. The presence of colour was therefore a salient property of the experiment. Participants looked more at colour competitors than unrelated distractors on hearing the target word in the colour trials but did not in the greyscale trials. In other words, when hearing ‘spinach’ they looked at the green frog but not the greyscale frog.

Experiment 2 is therefore consistent with the results of Experiment 1 in that it suggests that language-mediated eye movements are only influenced by colour relations between spoken words and visually displayed items if colour is present in the immediate visual environment. This further supports the notion that language users do not automatically simulate colour and that colour representations are not activated routinely. Experiment 2 is in line with the interpretation that colour representations are only retrieved and affect behaviour such as semi-automatic eye gaze when the context strongly supports or encourages the use of such information.

## Experiment 3

Experiment 3 was designed in the same way as Experiment 2 except that the visual display was removed at the sentence onset (i.e. after a long preview). This final experiment was conducted to examine whether the continued presence of colour in the immediate visual environment is necessary for the observation of colour-mediated eye movements. Eye movements directed towards the now blank screen were recorded as the sentence unfolded, the so-called blank screen paradigm (cf. Spivey & Geng, 2001; Altmann, 2004). We used this paradigm because previous studies (e.g., Gaffan, 1977; Sands & Wright, 1982; Wolfe, 2012) have shown that people can search their memory for pictures that are no longer present. Specifically, participants direct eye movements towards locations that were previously occupied by target objects even though this was completely unnecessary for the task (e.g., Altmann, 2004; Dell’Acqua, Sessa, Toffanin, Luria, & Jolicoeur, 2010; De Groot, Huettig, & Olivers, 2016; Hoover & Richardson, 2008; Johansson & Johansson, 2014; Laeng & Teodorescu, 2002; Richardson & Kirkham, 2004; Richardson & Spivey, 2000; Spivey & Geng, 2001; Theeuwes, Kramer, & Irwin, 2011). These so-called “looks at nothing” are typically interpreted as showing that participants have formed episodic memory traces in which the visual object identities are bound to their (previous) locations. If spoken language refers to the (previously fixated) target object then this is assumed to lead to retrieval of its original location, in turn triggering an eye movement (Ferreira, Apel, & Henderson, 2008; Richardson, Altmann, Spivey, & Hoover, 2009). The blank screen paradigm therefore represents another strong test for understanding the task dependency of embodied language processing. If colour information is routinely accessed and simulated, then we should observe evidence of it with the blank screen method.

### Method

#### Participants

A new sample of 30 Dutch native speakers from the MPI for Psycholinguistics database were invited, and after signing the informed consent form, took part in Experiment 3 for monetary compensation. They had normal vision, or otherwise wore soft contact lenses or glasses.

#### Materials and design, procedure and data analysis

Language and visual materials were kept the same as in Experiment 2, as well as the experimental design, thus, colour and greyscale trials were intertwined within experimental lists. The procedure was also identical, except for the stimuli presentation timing. Unlike the previous experiments, where the visual display and the spoken sentences were presented concurrently, in Experiment 3 the visual context was presented for 3000 ms and then disappeared with the onset of corresponding sentences. Consequently, the eye record reflects looks to an empty white screen were critical objects used to be. The data analysis approach is the same as that in the previous experiments. Data files and script are available at https://osf.io/853nk/.

#### Results and discussion

**Figure 5:**
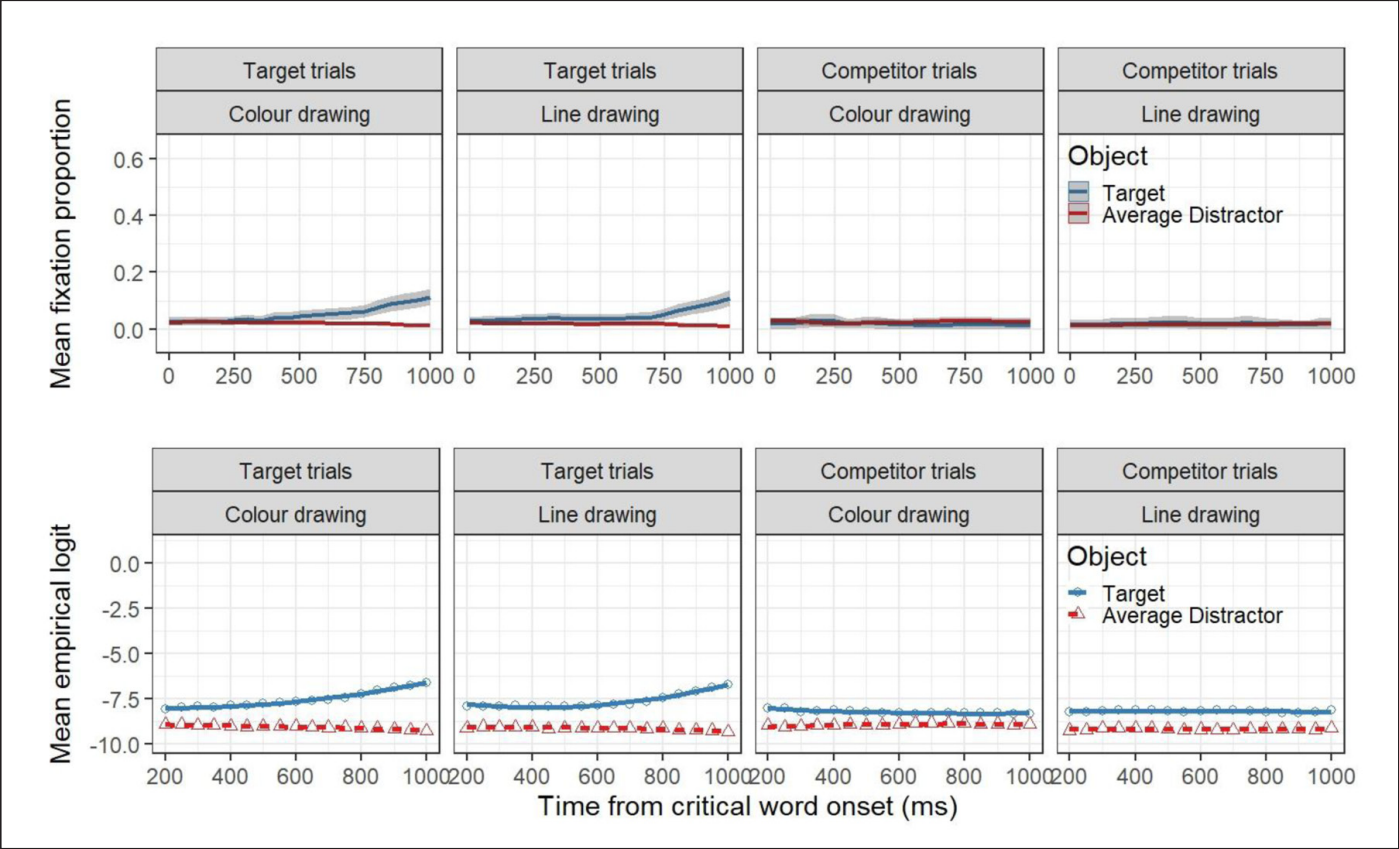
Upper panels: Mean fixation proportion as a function of trial type, aggregated in time steps of 50 ms. The plots are time-locked to the onset of the critical word. Blue and red lines represent looks to the target and the average distractor, respectively and the shaded areas represent within-subject adjusted 95% confidence levels, calculated by participants. Lower panels: GCA model fit (lines) of empirical logit (points) to the target and the average distractors as a function of trial type and experimental condition.

The upper panels in Figure 5 show four time-course plots, each of them depicting fixation proportions over a 1000 ms time window. The lower panels present empirical logit values together with the GCA model fit. In all graphs, blue lines represent the preference for critical objects and the red lines represent the same measure for the average distractors in the visual context. Grey shaded areas in the upper panels represent CIs around the mean proportion of fixations. The GCA model comparison resulted in the selection of a model that included the three first orthogonal polynomial terms (i.e., linear and quadratic, χ^2^ = 76.89, *df* = 18, *p* < .001).

Figure 5 shows that, as in the previous experiments, the proportion of fixations towards the location where the critical object used to be in the target condition (both in colour and greyscale condition) was higher relative to the average distractor. The graphs show that the region where the critical object used to be received more overt attention compared to where the distractors were previously shown. However, competitor trials showed a different pattern of effects: locations where critical objects used to be were not preferred compared to the previous distractor locations during the 1000 ms time window.

Table 4 shows the results of the GCA analysis. We found main effects of object, trial type and colour trials, reflecting the overall differences between targets and average distractors, between the competitor and target trials, and between coloured and greyscale trials. Moreover, the GCA shows reliable interaction effects between object and trial type, between object and colour condition, as well as between the linear term and object and trial type, and between the quadratic term and object and trial type. The first two interactions are evidence for a larger difference between targets and average distractors on target trials, as well as on colour trials (likely brought about by the target trials, where the colour conditions produce a larger target preference). In turn, the four subsequent two-way interaction effects reflect a more linear time course of the average distractors relative to targets (which assume a more quadratic time course), and the same tendency for competitor trials compared to target trials (which also take a more quadratic time course). We also found three-way interactions between the object, trial type and colour condition. Based on Figure 5 (lower panels), this interaction is likely to reflect a larger preference for the target on colour trials (vs. greyscale) on target trials (vs. no difference between the target and average distractors on competitor trials).

**Table 4:** Main and interaction effects in the quasi-logistic GCA mixed model analysis in Experiment 3.

	Estimate	se	t

(Intercept)	–8,52	0,11	–80,03*
Linear	0,30	0,26	1,16
Quadratic	0,15	0,11	1,37
Object	–0,59	0,01	–42,34*
Type	0,18	0,02	11,29*
Colour	0,05	0,01	3,39*
Object * Type	–0,17	0,01	–12,17*
Object * Colour	0,03	0,01	2,41*
Type * Colour	0,00	0,01	–0,09
Linear * Object	–0,39	0,18	–2,12*
Linear * Type	0,33	0,15	2,24*
Linear * Colour	–0,05	0,14	–0,36
Quadratic * Object	–0,19	0,09	–2,11*
Quadratic * Type	0,14	0,07	2,10*
Quadratic * Colour	0,00	0,08	–0,03
Object * Type * Colour	–0,04	0,01	–2,99*
Linear * Object * Type	–0,56	0,06	–8,67*
Linear * Object * Colour	–0,01	0,06	–0,19
Linear * Type * Colour	0,11	0,06	1,78
Quadratic * Object * Type	–0,17	0,06	–2,75*
Quadratic * Object * Colour	0,01	0,06	0,11
Quadratic * Type * Colour	–0,06	0,06	–1,03
Linear * Object * Type * Colour	–0,11	0,06	–1,95
Quadratic * Object * Type * Colour	0,08	0,06	1,45

Finally, we found reliable interactions between the linear term, object and trial type, and between the quadratic term, object and trial type. These three-way interactions can be interpreted as reflecting that the more linear and quadratic time course of the average distractors and targets (respectively), is more pronounced for target trials relative to competitor trials.

In sum, in the target trials, participants looked more at the locations where the targets (rather than the distractors) had been previously shown. This occurred as the target words acoustically unfolded, which shows that the blank screen set-up worked as expected. Crucially, in the competitor trials, such an effect was not observed: the locations where the colour competitors had previously been shown did neither in colour nor greyscale trials receive any increased attention.

## General Discussion

A central challenge for embodiment research is to better understand the task dependency of embodied language processing. Here we tested the influence of colour representations during language processing in three visual-world eye tracking experiments. The method is particularly well suited to investigate this issue because the availability of task-relevant visual input can be manipulated. Applying the visual-world eye-tracking method allowed us to make use of semi-automatic eye gaze behavior that has been investigated in a great number of studies. Specifically, we used the phenomenon that when participants hear a word that refers to a visual object or printed word, they quickly direct their eye gaze to objects or printed words which are similar (e.g. semantically or visually) to the heard word. We applied a look and listen task which previously has been shown to be very sensitive to such relationships between spoken words and visual items.

In Experiment 1, on competitor trials, participants listened to sentences containing a critical target word associated with a prototypical colour (e.g. ‘…spinach…’) as they inspected a visual display with four words printed in black font. One of the four printed words was associated with the same prototypical colour (e.g. green) as the spoken target word (e.g. FROG). On competitor trials, the spoken target word did not have a printed word counterpart (SPINACH was not present in the display). In target trials (70% of trials) the target was present in the display and attracted significantly more overt attention than the unrelated distractors. In competitor trials, colour competitors were not looked at more than the distractors. In Experiment 2 the printed words were replaced with line drawings of the objects. In order to direct the attentional focus of our participants towards colour features we used a within-participants counter-balanced design and alternated colour and greyscale trials randomly throughout the experiment. Therefore, on one trial our participants heard a word such as ‘spinach’ and saw a frog (coloured in green) in the visual display. On the next trial however they saw a banana (in greyscale) on hearing ‘canary’ (bananas and canaries are typically yellow). The presence (or absence) of colour was thus a salient property of the experiment. Participants looked more at colour competitors than unrelated distractors on hearing the target word in the colour trials but not in the greyscale trials, i.e. on hearing ‘spinach’ they looked at the green frog but not the greyscale frog. Experiment 3 was identical to Experiment 2, except that the visual display was removed at the sentence onset, after a longer preview. This experiment examined whether the continued presence of colour in the immediate visual environment was necessary for the observation of colour-mediated eye movements. Eye movements directed towards the now blank screen were recorded as the sentence unfolded (cf. Spivey & Geng, 2001). In the target trials, participants looked significantly more at the locations where the targets, rather than the distractors, had been previously presented as the target words acoustically unfolded. In the competitor trials, the locations where the colour competitors had previously been presented did not attract increased attention (neither in colour nor greyscale trials).

The results of all experiments presented in this study are exceedingly clear and converge on the same conclusion, namely, that language-mediated eye movements are only influenced by colour relations between spoken words and visually displayed items if colour is present in the immediate visual environment. An important advantage of the paradigm used here is that is very well suited to investigate the task dependency of embodied language processing, specifically, whether colour representations are routinely activated in context, or, whether such an influence emerges only when the context strongly supports or encourages their involvement.

An alternative interpretation of the present study is that when colour information is absent from the visual display, colour representations nonetheless get automatically activated and simulated in response to hearing ‘spinach’ but that language-mediated eye movements fail to capture such activation or simulation. Such an account is very unlikely to be correct. This is because a large amount of experimental work has shown that spoken language guides visual orienting without volitional control to visually concurrent objects which only partially match the representations activated by the spoken word and visual objects (e.g. semantically, visually, etc., see Mishra et al., 2013, for a detailed discussion). Such language-mediated eye movements are fast, unconscious, and largely overlearned and fit most of the criteria of an automatic process (cf. Logan, 1988; Moors & De Houwer, 2006). The work within the visual world and visual search paradigms strongly suggest that some prior conditions need to be met for language to be able to drive eye movements (Mishra et al., 2013). These conditions are to actively listen to the relevant speech and a predisposition to make eye movements (i.e. to look around rather than to focus on one location). Importantly, the integration of language with oculomotor behaviour tends to be unstoppable once these conditions are met (cf. Salverda & Altmann, 2011). It is therefore very unlikely that colour representations were activated and simulated in the present study in the cases in which eye movements did not reveal looks to colour competitors.

It is however certainly the case that the interactions between language and visual processing in the present experiments are complex. It is noteworthy that the conditions in the present experiments in which we did not observe shifts in overt attention to the colour competitor are the ones that, in addition to perceptually simulating the colour of the spoken word itself, either (i) require a second perceptual simulation to take place in parallel (to access the colour of a written-word competitor or a non-coloured pictorial competitor, respectively - see Experiments 1 and 2) or (ii) involve the additional operation of retrieving the competitor’s colour from memory (see ‘blank screen’ Experiment 3). The (colour) language – (colour) vision link is obviously tighter in conditions in which only one mental operation has to be performed (access or simulation of the spoken word’s typical colour) than in conditions where two mental operations need to be performed and coordinated with one another (access or simulation of the spoken word’s colour on the one hand and simulation (or some other form of memory-retrieval) of the competitor’s colour on the other. A very relevant experiment in this regard was conducted by Yee, Huffstetler, and Thompson-Schill (2011). They observed that on hearing ‘frisbee’ participants in a similar visual world experiment looked preferentially at a triangular piece of pizza. In other words, their participants (on seeing the slice of pizza) retrieved that pizza’s are typically round (like frisbees) which triggered overt attention shifts to the triangular pizza piece. Given their results about ‘non-depicted shape’ it is therefore very unlikely that in our present study participants should find it difficult to retrieve the ‘non-depicted colour’ (e.g. green) of the colour competitor (e.g. the greyscale frog). Nevertheless, we acknowledge, that the exact dynamics of language – vision interactions which are tapped in visual world experiments are complex and require further careful experimentation (see Huettig, Hartsuiker, & Olivers, 2011; Magnuson, 2019; Smith, Monaghan, & Huettig, 2017; for further discussion). It is important to emphasize though here again that the visual world paradigm is a good proxy for many real-world situations in which language guides our attention around the visual world. We often hear others tell us to “mind the step”, to “look at the beautiful flower”, or we receive directions in an unknown neighbourhood via mobile phones. In all such situations visual processing of our surroundings is tightly coordinated with our linguistic processing. These are therefore situations in which embodied language processing, presumably, would be most advantageous.

Our findings thus provide strong constraints for embodied theories of language processing. The present results fit best with the notion that the main role of perceptual representations in language processing is *not* to take part in ‘routine simulation’. It suggests that in absence of an immediately relevant visual environment, for instance while reading a novel, perceptual (i.e. ‘embodied’) simulations if they occur, may well be rather impoverished. Such an account of ‘impoverished simulation’ is very much compatible with the central tenets of good-enough processing theory of sentence processing (Ferreira et al., 2002, Ferreira & Patson, 2007). Readers or listeners do not necessarily activate complete representations (e.g. syntactic, semantic, visual, etc.) of an unfolding utterance or sentence. Indeed, they may often not activate content in great detail, rather, they often activate (or simulate) ‘just good enough representations’ to get the gist of an utterance.

The results from the present study may appear to conflict with results investigating colour relations with the sentence-picture verification paradigm. Interestingly, such studies found effects in different directions, Connell (2007) found that pictures *mismatching* a colour implied in sentences facilitated reaction times in sentence-picture verification whereas Mannaert, Dijkstra and Zwaan (2017; see also Zwaan & Pecher, 2012) observed that pictures *matching* a colour implied in sentences facilitated responses. Crucially, both sets of results were interpreted as revealing embodied language processing (see Connell & Lynott, 2012; Guerra & Knoeferle, 2018 for a discussion). This divergence in findings highlights, we believe, the need to assess embodiment using a diverse set of methods. We invite researchers to attempt to replicate the present results and to explore them further using other paradigms. Converging evidence from other methods will ultimately show whether our interpretation of the data is on the right track or not.

To conclude, we conducted three visual world experiments to understand the task dependency of embodied language processing. Specifically, we explored whether colour representations are activated routinely during online language understanding. Our results do not fit with routine activation but suggest that the role of perceptual representations in language processing may be a different (but nevertheless very important) one, namely to contextualize language in the immediate environment, connecting language to the here and now. Such an interpretation, arguably, also straightforwardly fits with many seminal ‘embodiment effects’ during language processing (e.g. the ones based on sentence picture verification in which participants have to verify whether a pictorially presented concrete object was mentioned in the preceding sentence). We challenge proponents of the ‘strong view’ that *perceptual simulation routinely occurs during language processing in absence of visual input* to present such evidence (i.e. evidence from tasks that do not involve the presentation of visual stimuli). Note that fMRI evidence of partial activation of sensory (e.g. colour) representations during language processing does not provide such unequivocal evidence (see Coltheart, 2013; Rugg & Thompson-Schill, 2013, for further discussion). In short, we propose that future research (including studies using other paradigms) should focus more directly than current embodied language processing research on the role of perceptual representations that *contextualize language* in the immediate environment.

## Data Accessibility Statement

Data files and script are available at https://osf.io/853nk/.
